# Cdk1 activity acts as a quantitative platform for coordinating cell cycle progression with periodic transcription

**DOI:** 10.1038/ncomms11161

**Published:** 2016-04-05

**Authors:** Gabor Banyai, Feriel Baïdi, Damien Coudreuse, Zsolt Szilagyi

**Affiliations:** 1 Department of Medical Biochemistry and Cell Biology, University of Gothenburg, Medicinaregatan 9A, PO Box 440, 41390 Gothenburg, Sweden; 2 SyntheCell team, Institute of Genetics and Development of Rennes, CNRS UMR 6290, 2 Avenue du Pr. Léon Bernard, 35043 Rennes, France

## Abstract

Cell proliferation is regulated by cyclin-dependent kinases (Cdks) and requires the periodic expression of particular gene clusters in different cell cycle phases. However, the interplay between the networks that generate these transcriptional oscillations and the core cell cycle machinery remains largely unexplored. In this work, we use a synthetic regulable Cdk1 module to demonstrate that periodic expression is governed by quantitative changes in Cdk1 activity, with different clusters directly responding to specific activity levels. We further establish that cell cycle events neither participate in nor interfere with the Cdk1-driven transcriptional program, provided that cells are exposed to the appropriate Cdk1 activities. These findings contrast with current models that propose self-sustained and Cdk1-independent transcriptional oscillations. Our work therefore supports a model in which Cdk1 activity serves as a quantitative platform for coordinating cell cycle transitions with the expression of critical genes to bring about proper cell cycle progression.

## Abstract (web summary)

Cell proliferation is regulated by cyclin-dependent kinases (Cdks) and relies on periodic gene cluster expression according to cell cycle phases. Here the authors use a synthetic regulatable Cdk1 module to demonstrate that periodic expression is governed by quantitative changes in Cdk1 activity.

## Graphical abstract

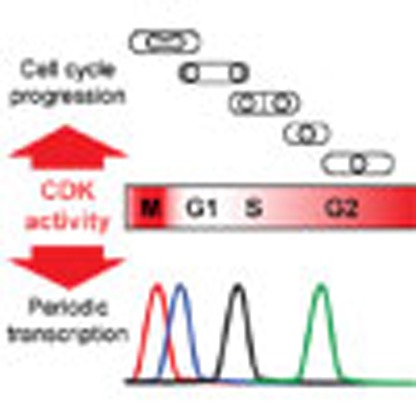

The eukaryotic cell cycle is a highly regulated process that relies on intricate mechanisms to ensure faithful duplication and segregation of the genetic material. The succession of cell cycle phases coincides with and depends on the periodic transcription of specific sets of genes, a phenomenon that is conserved among all eukaryotes tested to date. Genome-wide studies in models ranging from yeast to human cells have led to the identification of a substantial pool of periodic genes that have been clustered according to their peak time of expression and associated with M (mitosis), G1, S (DNA replication) or G2 (refs 1, 2, 3, 4). Although the overall list of genes belonging to this transcription program differs between species, a conserved core of periodic gene networks has now been revealed, highlighting its evolutionary importance^5^.

Initial insights into the control of cell cycle oscillations in gene expression were derived from studies in the budding yeast *Saccharomyces cerevisiae* (*S. cerevisiae*)^3^,^5^,^6^. In this system, the periodic transcriptional program was proposed to rely on the fact that each gene cluster contains the transcription factors (TFs) that control the expression of the following cluster. Moreover, the observation that oscillations in gene expression still occurred in G1-arrested cells suggested that the established circuit of serially induced TFs is sufficient to generate periodicity^7^,^8^. Even in the absence of all Cdk1 activities, ∼66% of cell cycle-regulated transcripts continued to be periodic^8^. From these results, a model emerged in which the kinetics of the transcriptional oscillations play a key role in bringing about the succession of cell cycle events. In this case, the major regulators of cell cycle progression, the cyclin-dependent kinases (Cdks), would act as effectors of the transcriptional oscillator: for example, entrainment of cyclin expression to this system could sequentially trigger the G1/S and G2/M transitions through the association of various cyclins with Cdks^8^. However, it remains unknown whether this architecture represents a conserved mode of regulation of periodic transcription among eukaryotes.

In the fission yeast *Schizosaccharomyces pombe* (*S. pombe*), cell cycle-associated periodic transcription involves ∼500 genes^9^,^10^,^11^,^12^ and already appears to differ from what is observed in *S. cerevisiae*. The MCB-binding factor (MBF) complex regulates M/G1 and early G1 genes that encode for proteins involved in both the G1/S transition and the duplication of the genome during S phase. Later in G1, a set of periodic genes enriched in factors that are linked to cell separation after mitosis is regulated by the Ace2 transcription factor^9^,^13^, while expression of the histone genes in S phase depends on the GATA transcription factor Ams2 (ref. 14). Apart from those controlled by MBF, a number of genes induced at mitosis are regulated by the PCB-binding factor (PBF) complex^3^. Despite this apparently detailed knowledge of the regulation of periodic expression, little is known about what controls the transcription of the largest fractions of the G1, S and G2 clusters. In addition, the budding yeast TF network seems to be only partially present in *S. pombe*. For instance, although PBF regulates *ace2* expression, which in turn activates the G1 genes, MBF targets are controlled independently of the PBF-dependent genes^3^. Furthermore, *ams2* expression relies on MBF, but there is no evidence for a direct link between MBF-dependent transcription and the expression of histone genes. This suggests that the coupling of cell cycle progression with periodic transcription may be governed by a different logic in this system. Understanding these mechanisms may therefore lead to novel models for the coordination of the processes linked to cell proliferation in eukaryotes.

A central player in cell cycle progression is the conserved Cdk1 protein, the predominant member of the Cdk family. In the budding and fission yeasts, Cdk1 controls both the G1/S and G2/M transitions^15^. As mentioned previously, transcriptional oscillations still occur in *S. cerevisiae* cells that are arrested in their cycle, indicating that this periodicity is phase-independent and that Cdk activity acts in parallel or downstream of the transcriptional program. However, the ability to re-program the fission yeast cell cycle network and alter the sequence of cell cycle events simply by artificially changing Cdk1 activity^16^ raises the possibility of a tight link between cell cycle phases and expression of critical periodic genes.

To address this, we take advantage of a recently described minimal cell cycle network in fission yeast in which Cdc2/Cdk1 is fused to the B-type cyclin Cdc13 (ref. 16). The level of this module oscillates through cycles of synthesis/degradation rather than strong cell cycle-regulated expression^9^. Importantly, its kinase activity can be finely regulated to alter the progression of the cell cycle at all phases of the process. This previously allowed us to demonstrate that cell cycle transitions are primarily driven by quantitative changes in the levels of a single qualitative Cdk activity (that is, the function of Cdk1 in association with a specific cyclin), a model that appears to be conserved in more complex eukaryotes^17^,^18^,^19^. Here we use this system to dissect the interplay between Cdk1 activity and periodic transcription. We first demonstrate that periodic gene expression in fission yeast does not show cell cycle-independent oscillations, regardless of the phase in which cells are arrested. We then uncover that the coupling between periodic transcription and cell cycle stages relies on a quantitative response to Cdk1 activity levels. We further show that cell cycle events neither participate in nor interfere with this transcriptional program, provided that cells are subjected to the appropriate Cdk1 activity levels. Our results challenge the widely accepted model of self-sustained, Cdk1-independent transcriptional oscillations that emerged from studies in budding yeast. We therefore propose that the regulation of transcription throughout the cell cycle is tightly linked to dynamic changes in the activity levels of the cyclin-dependent kinases. This may represent a conserved architecture in which Cdks act as a quantitative platform for coordinating critical cell cycle events with periodic transcription, providing new insight into the systems that control the proliferation of eukaryotic cells.

## Results

### Loss of periodic transcription in G1-arrested cells

**Figure 1:**
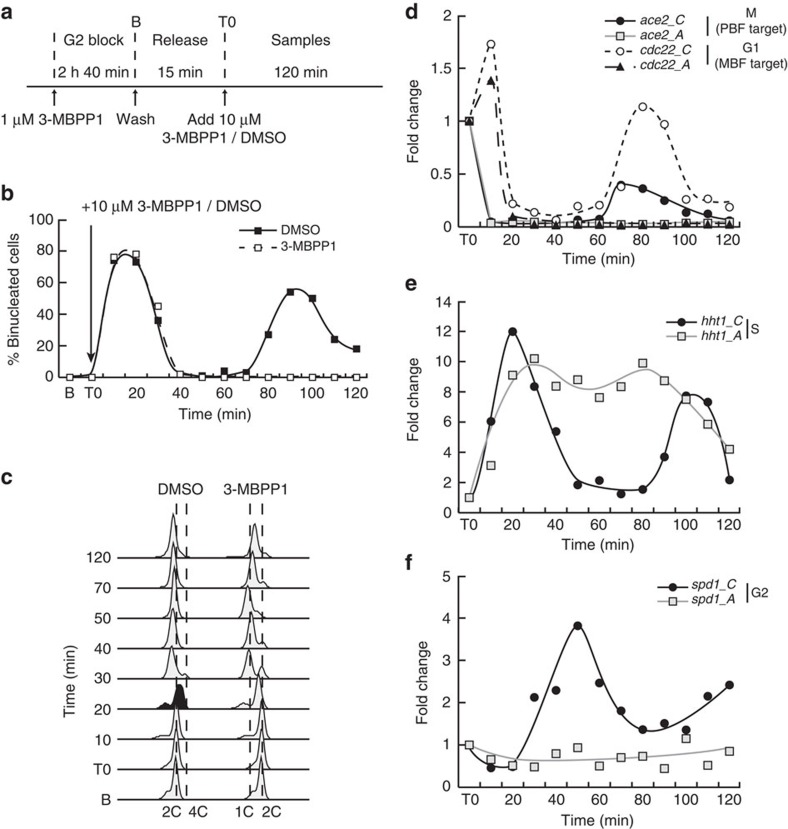
Periodic transcription in G1-arrested fission yeast cells. (**a**) Schematic representation of the experimental procedure. Inhibitor-sensitive minimal cells were synchronized in G2 by addition of 1 μM 3-MBPP1 for 2 h 40 min. Cultures were washed to allow cells to resume their cycle, progress through the metaphase to anaphase transition (15 min, Release), and finally treated with 10 μM 3-MBPP1 to induce a G1 arrest (DMSO was used as a control)^16^. Samples were then taken every 10 min for 120 min and gene expression changes were assessed. (**b**) Percentage of binucleated cells in **a**. Both control and inhibitor-treated cells initially undergo mitosis. However, cells subsequently treated with inhibitor at T0 remain blocked in G1 (see **c**) and do not progress to the next mitosis. *n*>100 at each time point. (**c**) DNA content analysis of cells in **a**. Although control cells progress through S phase (black profile) and cytokinesis, inhibitor-treated cells accumulate in G1 prior to S phase with a 1C DNA content (see Methods section for the interpretation of flow cytometry profiles in fission yeast). (**d**–**f**). Changes in gene expression for the indicated genes in **a**. *C* and *A* refer to cycling (DMSO-treated) and arrested (inhibitor-treated) cells, respectively. Fold changes are normalized to actin RNA levels and represented relative to the values at T0 (set to 1). See Supplementary Fig. 1 for additional representative genes.

To understand the interaction between cell cycle progression and periodic expression in the context of the fission yeast TF network, we assessed the transcriptional program upon inhibition of Cdk1 activity in G1, which prevents DNA replication. Indeed, cell cycle-independent transcriptional oscillations were initially revealed in budding yeast cells arrested in G1 by inhibition of mitotic cyclin function or using a temperature-sensitive allele of Cdk1/Cdc28 (refs 7, 8). To control cell cycle progression in our model, we used minimal cells in which the activity of an engineered cyclin B-Cdk1 fusion can be altered by addition of the small molecule inhibitor 3-MBPP1 (ref. 16). Synchronous cultures were allowed to progress through mitosis and subsequently treated with high concentrations of 3-MBPP1 to arrest cell cycle progression in G1 (ref. 16) (Fig. 1a–c). Changes in transcript levels of genes representative of the different periodic clusters were then measured and compared with those in cycling cells.

We first determined the behaviour of the mitotic cluster by analysing the PBF target gene *ace2*. As expected, its expression decreased as cycling cells exited mitosis and entered early G1 (Fig. 1d). Similar dynamics were observed in inhibitor-treated cells, consistent with the fact that Cdk1 activity is already strongly reduced at the time of inhibitor treatment due to APC-dependent degradation of the cyclin, an essential step in mitotic exit. Importantly, while an additional peak in *ace2* expression was observed at the next mitosis in cycling cells (Fig. 1b,d), its levels remained low in G1-arrested cells throughout the time course. The MBF-target gene *cdc22* (G1 cluster) peaked later but subsequently behaved similarly to *ace2*, with no second induction in inhibitor-treated cells (Fig. 1d). In contrast, their budding yeast orthologues, ACE2 and RNR1, are still periodically expressed in the absence of all Cdk1 activities^8^. Next, to assess the behaviour of the S-phase cluster, we focused on histone genes, which are among those with the highest induction amplitude^4^,^12^ and are commonly used as markers for this cluster. *hht1* was periodically expressed, peaking in cycling cells at the time of DNA replication (Fig. 1c,e). Intriguingly, *hht1* expression was also induced in G1-arrested cells with similar kinetics and levels as in the control, but the transcript levels remained high for most of the time course before slightly decreasing towards the end of the experiment. This suggests that once cells have passed mitosis, commitment to DNA replication itself is not critical for induction of this cluster. However, its periodic behaviour is contingent on cell cycle progression through S and entry into G2. Finally, the G2 gene *spd1* peaked during G2 in the cycling culture as anticipated (Fig. 1b,f) and remained low throughout the experiment in G1-arrested cells. Taken together, these results suggest that cell cycle-independent periodic oscillations in transcription are absent in G1-arrested fission yeast cells.

### Loss of periodic transcription in G2-arrested cells

**Figure 2:**
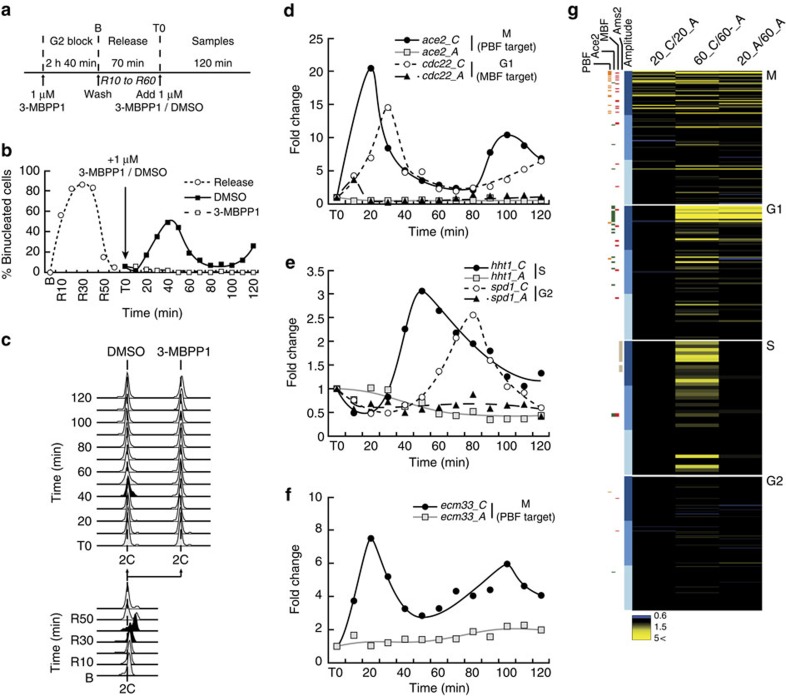
Periodic transcription in G2-arrested fission yeast cells. (**a**) Schematic representation of the experimental procedure. Inhibitor-sensitive minimal cells were synchronized in G2 with 1 μM inhibitor (3-MBPP1) for 2 h 40 min as described^16^. Upon washing off the inhibitor, cells resumed their cycle (Release), progressing through mitosis, G1, S and entering the next G2 after ∼70 min (see **b** and **c**). The culture was subsequently treated with 1 μM 3-MBPP1 (T0), blocking the onset of the next mitosis. DMSO-treated cells were used as a control. Samples were then taken every 10 min for 120 min, and gene expression changes were assessed. (**b**) Percentage of binucleated cells in **a**. Unlike the control culture, cells treated with inhibitor at T0 do not undergo mitosis. Release is as in **a**. *n*>100 at each time point. (**c**) DNA content analysis of cells in **a**. At T0, cells have undergone DNA replication (black profile, bottom) and have entered G2. Subsequently, control cells undergo a second round of replication after 40 min (black profile, top), while inhibitor-treated cells are blocked in G2. Note that the reduced synchrony in the second cell cycle makes the next S phase more difficult to monitor by flow cytometry (see DMSO profiles). (**d**–**f**) Changes in gene expression for the indicated genes in **a**. *C* and *A* refer to cycling (DMSO-treated) and arrested (inhibitor-treated) cells, respectively. Fold changes are normalized to actin RNA levels and represented relative to the values at T0 (set to 1). (**g**) Heatmaps reflecting the differences in expression levels (ratios between conditions) for periodic genes between cycling (*C*) and arrested (*A*) cells at the indicated time points. Included are ∼450 periodic genes as described in Cyclebase (Supplementary Data 1), sorted by induction amplitude^4^,^12^. Coloured boxes on the left show the known targets of PBF, Ace2, MBF and Ams2. Within each cluster, shades of blue indicate three groups according to their transcription amplitude, each containing the same number of genes. Darker blue corresponds to higher fold inductions. See Supplementary Table 1 for the percentages of genes above the cutoffs for the ratios in expression between the cultures.

Autonomous transcriptional oscillations may require a specific trigger within the TF network that could be associated with discrete phases of the cell cycle. Interestingly, the organization of the budding yeast cell cycle is G1-driven, while fission yeast proliferation relies on strong regulation at the G2/M transition. This prompted us to assess if oscillations in transcription occur in G2-arrested fission yeast cells. Synchronous cultures were allowed to proceed through mitosis, G1 and S, eventually entering the next G2 (Fig. 2a, T0). Half of the culture was then arrested in G2 using 1 μM 3-MBPP1, whereas the other half kept cycling (Fig. 2b,c). This allowed for a direct comparison of the transcription of periodic genes between G2-arrested and proliferating cells.

As seen in G1-arrested cells, periodic induction of *ace2* and *cdc22* in M and G1, respectively, was strongly dependent on cell cycle progression (Fig. 2d), as no activation was detected in inhibitor-treated cells. In contrast to the G1 experiment, *hht1* (S) remained at low levels in arrested cells, consistent with a repression of its expression in G2 (Fig. 1e). We finally found that the G2 gene *spd1* was not induced, despite cells being arrested in G2 (Fig. 1e). This suggests that the timing of inhibitor treatment occurred after *spd1* levels had declined (compare Figs 1 and 2). Preventing another cycle through M, G1 and S into the next G2 therefore precluded another round of *spd1* induction.

To address whether these results on representative genes reflect the global behaviour of the corresponding clusters, we performed genome-wide analyses in the same experimental setup, assessing periodic transcription of the M (20 min) and S (60 min) clusters (Fig. 2g). Genes within each cluster were grouped in three categories according to the amplitude of their induction^4^,^12^ (Fig. 2, legend). Our results showed that 53% of the mitotic genes of the high expression group behaved similarly to *ace2* when comparing cycling and arrested cells at 20 min (20_C/20_A in Fig. 2g and Supplementary Table 1, cutoff for the ratio cycling/arrested cells is 1.5). However, other high-amplitude genes, such as *ecm33*, did not make the cutoff, suggesting that they might also be activated in the arrested cells. Nevertheless, when testing *ecm33* by qPCR, we found no induction in inhibitor-treated cells (Fig. 2f). This prompted us to reduce the cutoff to 1.15, a value just below that measured for *ecm33* (1.19). With this threshold, 82% of the high amplitude mitotic genes showed no periodic transcription upon cell cycle arrest (Supplementary Table 1). As expected by the timing of the cell cycle phases in these experiments and our qPCR data, expression of G1, S and G2 genes did not change in the 20_C/20_A analysis (Fig. 2g; Supplementary Table 1). This again contrasts with the 66% of cell cycle-regulated transcripts that continue to oscillate in the absence of all Cdk1 activities in budding yeast^8^.

At the 60 min time point, we observed that 85% of the entire set of S-phase genes showed significantly lower expression in the arrested cells (60_C/60_A, Fig. 2g and Supplementary Table 1), consistent with the *hht1* qPCR results. We also noted that a significant percentage of M and G1 genes still made the cutoff (Supplementary Table 1), in contrast with the decrease in *ace2* and *cdc22* expression detected by qPCR. However, this likely results from a reduction in their basal transcript levels in the arrested cells during the experiment (see 20_A/60_A in Fig. 2g, Supplementary Table 1), a phenomenon that was less pronounced for the S and G2 clusters. Finally, a lower percentage of the G2 genes passed the threshold, even after 60 min, due to their later peak time (see *spd1* in Fig. 2e).

Together with the data obtained with G1-blocked cultures, this suggests that the transcriptional program in fission yeast fundamentally differs from that in budding yeast, as periodic genes do not freely oscillate in cells arrested in the cycle. This could result from either the induced pause in cell cycle progression or a more direct link between changes in Cdk1 activity and transcriptional oscillations.

### Periodic transcription is linked to cell cycle progression

**Figure 3:**
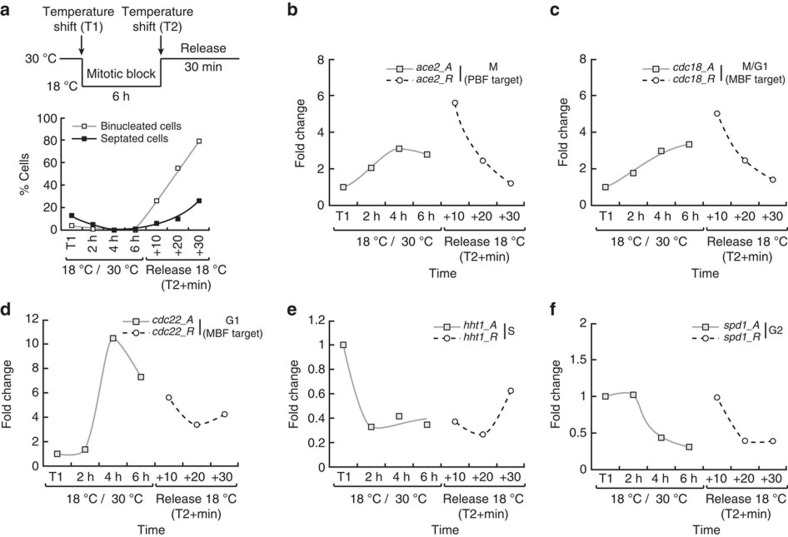
Coupling of periodic transcription and cell cycle phases. (**a**) Top panel: schematic representation of the experimental procedure. Cultures of the *nda3-km311* cold-sensitive mutant^29^ grown at 30 °C were shifted to the restrictive temperature of 18 °C (T1) for 6 h (mitotic block) before being shifted back to the permissive temperature (Release, T2). Samples were taken at the indicated times during the mitotic block and after the release, and changes in expression of representative periodic genes were assessed. Bottom panel: percentage of binucleated and septated cells during the experiment (*n*=100 at each time point). (**b**–**f**) Changes in gene expression in **a** for the indicated genes. *A* and *R* refer to arrested (restrictive temperature) and released (30 °C upshift) cultures, respectively. Fold changes are normalized to actin RNA levels and represented relative to the values at T1 (set to 1).

Our results indicate that periodic transcription during the fission yeast cell cycle is linked to its different transitions. We next tested whether (1) the periodicity in expression of these genes only depends on the onsets of specific cell cycle events, with their subsequent decrease in transcription relying on a timer (trigger-timer process), or (2) the induction and downregulation of a gene cluster are respectively coupled to entering and exiting associated cell cycle phases, as suggested by *hht1* in G1-arrested cells (Fig. 1e). To address this, we assessed the transcription of representative genes when cells are temporarily blocked in mitosis, using a cold-sensitive mutant of the tubulin-encoding gene *nda3*. Switching the *nda3-km311* mutant cells to 18 °C activates the spindle assembly checkpoint, with cells arresting in early mitosis with high Cdk1 activity^20^. The culture was then shifted back to the permissive temperature, allowing for the resumption of cell cycle progression (Fig. 3a). Interestingly, we observed an accumulation of *ace2* transcripts (M cluster) during the arrest that only returned to basal levels once cells were allowed to proceed through mitosis (Fig. 3b). A similar behaviour was observed for the MBF targets *cdc18* and *cdc22* (Fig. 3c,d), suggesting that their initial induction and subsequent downregulation are also linked to mitotic onset and exit, respectively. The S and G2-associated genes *hht1* and *spd1* were repressed upon mitotic entry and remained at low levels during the block, as anticipated (Fig. 3e,f); however, the expression of *hht1* increased again 30 min after release, concomitant with cells progressing through G1 and S.

We therefore conclude that the periodicity in transcription of different clusters is dependent on sequential progression through cell cycle phases rather than operating on a trigger-timer model.

### Periodic transcription is imposed by Cdk1 activity changes

The link between cell cycle progression and periodic transcription may be brought about by different mechanisms. First, induction and downregulation of periodic genes could be strictly coupled to cellular events that are specific to each phase. Alternatively, oscillatory behaviour may directly derive from cell cycle-associated changes in Cdk1 activity levels rather than cell cycle progression *per se*. Indeed, Cdk1 activity is high at mitosis, strongly reduced upon mitotic exit, rises during G1 to pass the S-phase threshold and keeps increasing throughout G2 until the Wee1/Cdc25 feedback loop becomes activated^15^, at which point it reaches the mitotic threshold. These dynamic modulations in Cdk1 activity may provide an ideal quantitative framework for driving periodic transcription, regardless of cell cycle status.

**Figure 4:**
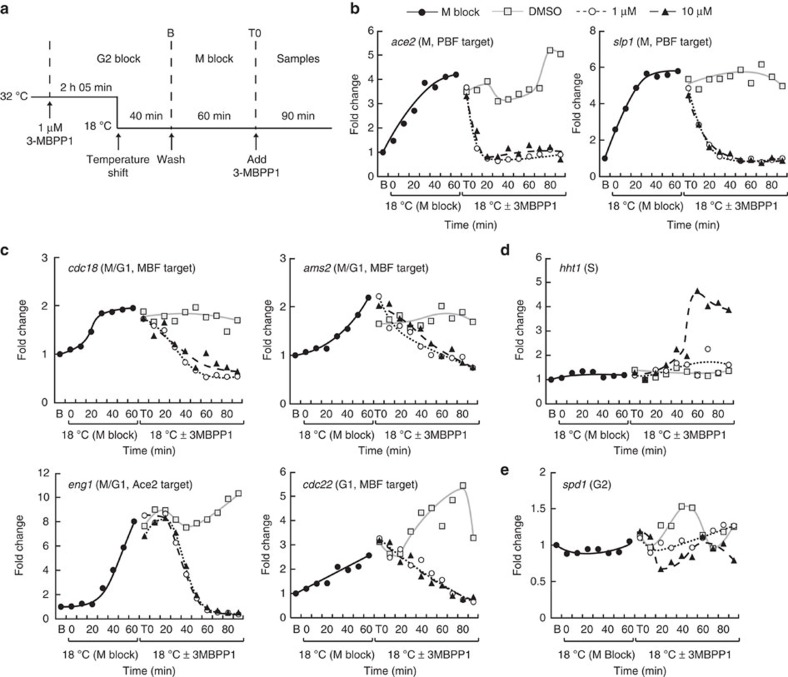
Periodic transcription and Cdk1 activity dynamics. (**a**) Schematic representation of the experimental procedure. Inhibitor-sensitive minimal cells carrying the *nda3-km311* cold-sensitive mutation were synchronized in G2 using 1 μM 3-MBPP1 at the permissive temperature (32 °C). Forty minutes before the end of the G2 block, the culture was shifted down to the restrictive temperature of 18 °C for the rest of the experiment to inactivate Nda3. Upon subsequent release from the inhibitor block, cells resumed their cycle but arrested in mitosis as a result of the *nda3-km311* mutation at this temperature (M block, also see Supplementary Fig. 2). After 60 min (T0), different concentrations of 3-MBPP1 were added to inhibit Cdk1 activity and samples were taken every 10 min for 90 min to assess changes in gene expression (DMSO was used as a control). (**b**–**e**) Changes in gene transcription in **a** for the indicated genes. Fold changes are normalized to actin RNA levels and represented relative to the values at B (set to 1). Legends in **c**–**e** are as in **b**.

To discriminate between these two possibilities, we uncoupled cell cycle progression from changes in Cdk1 activity. To this end, we used inhibitor-sensitive minimal cells carrying the *nda3-km311* mutation, which allowed us to block cells in mitosis with high Cdk1 activity via temperature shift^20^ and then repress Cdk1 using the 3-MBPP1 inhibitor while they remained arrested in M (Fig. 4a). In these conditions, cells experience a low Cdk1 activity without actually progressing through mitosis (Supplementary Fig. 2a,b).

Consistent with our previous results, the PBF targets *ace2* and *slp1* (M phase) as well as the MBF targets *cdc18*, *ams2* and *cdc22* (M/G1 and G1) were upregulated in mitotically blocked cells, although with different dynamics. Strikingly, downregulation of Cdk1 activity even with low inhibitor concentration (1 μM) was sufficient to strongly repress the expression of these genes to basal levels while cells remained in mitosis (Fig. 4b,c). Similar results were observed with other high amplitude mitotic genes (Supplementary Fig. 2c) and the Ace2 target *eng1* (Fig. 4c). These data demonstrate that the periodicity of M and G1 genes is solely driven by changes in Cdk1 activity.

The S phase histone genes *hht1* and *hhf1* remained low in the control, arrested cells (Fig. 4d and Supplementary Fig. 2d), indicating that G2/M levels of Cdk1 activity are sufficient to prevent the expression of this cluster. Interestingly, addition of 1 μM inhibitor had no effect on their expression, while 10 μM 3-MBPP1 resulted in rapid induction. Again, this was observed without resumption of mitosis. These data are in line with previous results showing that strong inhibition of Cdk1 activity in G2 through treatment with 10 μM inhibitor is necessary to allow entry into S without an intervening mitosis, while usage of 1 μM only maintains a G2 arrest^16^. Importantly, this difference in response between the two different inhibitor concentrations highlights the quantitative link between expression of periodic genes and Cdk1 activity levels.

For the G2 cluster, we did not observe striking changes in transcript levels of *spd1* (Fig. 4e). This is consistent with its low expression during mitosis and the inhibition of its induction when G2 cells are treated with 3-MBPP1 (Figs 1 and 2).

**Figure 5:**
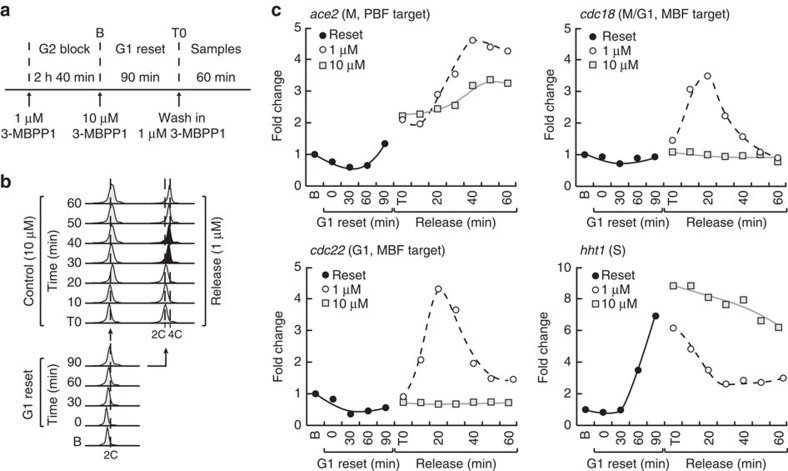
Periodic transcription upon bypass of cell cycle phases. (**a**) Schematic representation of the experimental procedure. Inhibitor-sensitive minimal cells were blocked in G2 by addition of 1 μM 3-MBPP1 for 2 h 40 min. The cultures were then treated with 10 μM 3-MBPP1 for 90 min to induce G1-reset without an intervening mitosis^16^. Subsequent release into 1 μM inhibitor at T0 (control cells were maintained in 10 μM 3-MBPP1) induces re-replication without an intervening mitosis^16^. Samples were collected during the reset and after the release at the indicated time points for assessing changes in gene transcription. (**b**) DNA content analysis of cells in **a**. Although control cells remain arrested with a 2C DNA content, cells released into 1 μM 3-MBPP1 enter S phase (black profiles), re-replicating their genome to a 4C DNA content. Note the shift of the cytometry profiles during the G1 reset, which results from cell elongation (see Methods). The dashed line indicating 2C DNA content was set based on the T0 profiles. (**c**) Changes in gene expression for the indicated genes in **a**. Fold changes are normalized to actin RNA levels and represented relative to the values at B (G2 block, set to 1).

We then tested the extent of this apparent lack of interplay between cell cycle events *per se* and periodic transcription by artificially altering the sequence of cell cycle phases, assessing the effect of bypassing mitosis on gene transcription. To this end, G2-blocked cells were treated with high concentrations of 3-MBPP1 (10 μM) and then released into lower concentrations (1 μM), allowing for G1 reset and re-replication of the genome without an intervening mitosis (Fig. 5a,b)^16^. Interestingly, we observed that compared with control cells maintained in 10 μM inhibitor, cells released in 1 μM 3-MBPP1 showed periodic induction of *cdc18* and *cdc22* (Fig. 5c), although with smaller fold changes than in our previous experiments (see Figs 1 and 2 and Discussion). Only a minor effect was detected for *ace2*. Consistent with the results in Fig. 4, elevated transcription of *hht1* on the initial strong inhibition of Cdk1 activity was repressed by the switch to 1 μM inhibitor (Fig. 5c). These data reinforce our conclusions that altering Cdk1 activity, even while bypassing cell cycle phases, is sufficient to regulate periodic transcription.

Our results strongly point towards a new model in which autonomous transcriptional oscillations do not entrain cell cycle progression. Instead, periodic transcription occurs as a result of the operation of the cell cycle machinery through dynamic changes in Cdk1 activity.

### Cell cycle transitions do not affect Cdk1-driven periodicity

Our data demonstrate that periodic transcription is closely linked to changes in Cdk1 activity, to the extent that modulating Cdk1 function in cell cycle-arrested cells is sufficient to reprogram transcription. However, these experiments were performed in arrested cells and therefore do not address the potential interplay between the expression of a set of genes during a given cell cycle phase and the onset of the next phase. For instance, together with the APC-dependent reduction in Cdk1 activity at mitotic exit, entry into S phase may further downregulate mitotic and G1 genes. This may represent an additional mechanism for ensuring the proper sequence of cell cycle events.

**Figure 6:**
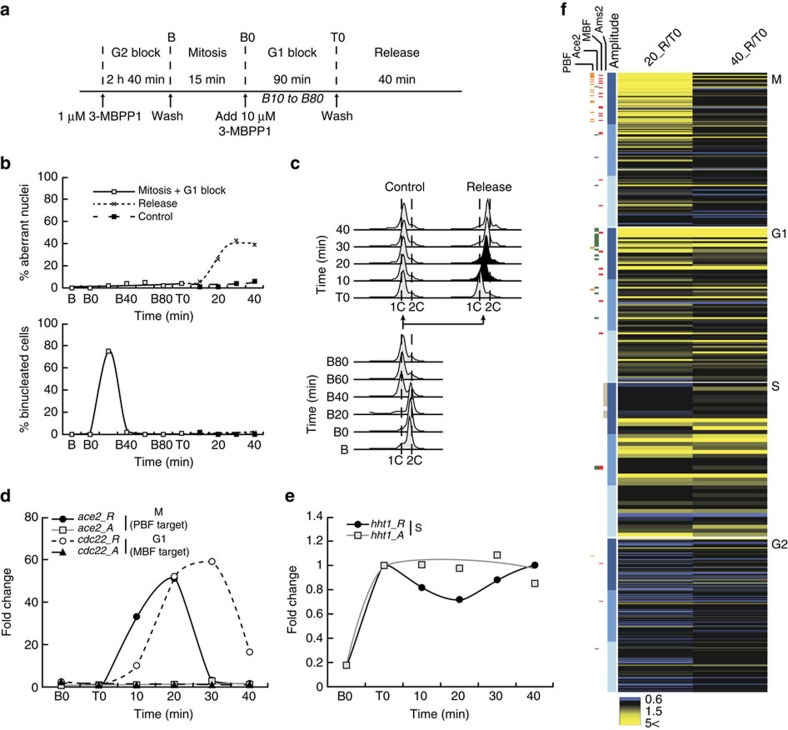
Interplay between cell cycle transitions and periodic transcription. (**a**) Schematic representation of the experimental procedure. Inhibitor-sensitive minimal cells were synchronized in G2 by addition of 1 μM 3-MBPP1 for 2 h 40 min, allowed to resume their cell cycle and progress through the metaphase to anaphase transition (mitosis, 15 min), and treated with 10 μM 3-MBPP1 (B0) for 90 min to induce a G1-arrest (G1 block). Cultures were released from the G1 block at T0, inducing overlapping S and M phases^16^ (cells maintained in 10 μM 3-MBPP1 were used as a control). Samples were collected at the indicated time points for assessing changes in gene expression. (**b**) Top panel: percentage of aberrant nuclei for cells in **a** (cells undergo DNA replication and mitosis simultaneously, giving rise to ‘cut' phenotypes). Bottom panel: corresponding percentages of binucleated cells. *n*>100 at each time point. (**c**) DNA content analysis of cells in **a**. Although the control cells remain blocked in G1 with a 1C DNA content, the released cells undergo rapid S phase (black profiles). (**d**,**e**) Changes in gene transcription for the indicated genes during the release in **a**. *R* and *A* refer to released (DMSO-washed) and arrested (maintained in 10 μM inhibitor after T0) cells, respectively. Fold changes are normalized to actin RNA levels and represented relative to the values at T0 (set to 1). (**f**) Heatmaps reflecting the differences in expression levels, comparing 20 and 40 min after the release with T0. Included are ∼450 periodic genes as determined in Cyclebase (Supplementary Data 2), sorted by transcription amplitude^4^,^12^. Coloured boxes on the left show the known targets of PBF, Ace2, MBF and Ams2. Within each cluster, shades of blue indicate three groups according to their transcription amplitude, each containing the same number of genes. Darker blue corresponds to higher fold inductions (also see Fig. 2). See Supplementary Table 2 for the percentages of genes above the ratio cutoff.

To address this, we analysed periodic transcription in cells induced to simultaneously undergo M and S^16^. This is achieved through blocking cells in G1 with high concentrations of inhibitor, allowing for the accumulation of inactivated fusion protein, followed by removal of the inhibitor, which results in a rapid increase in Cdk1 activity. The abnormally high activity generated in this context is sufficient to trigger both S (low-activity threshold) and M (high-activity threshold) at the same time, giving rise to aberrant nuclei (Fig. 6a–c). We found that both the PBF target *ace2* and the MBF target *cdc22* were highly induced upon mitotic entry, in contrast to cells in which low Cdk1 activity was maintained (Fig. 6d). Furthermore, both the periodicity (30–40 min) and the difference in the timing of peak expression between these two genes (10 min) were maintained as in cycling cells. This is consistent with our model in which quantitative changes in Cdk1 activity, which still occur in these experiments through the function of the APC at mitotic exit, control periodic transcription. Importantly, all of these events were concomitant with DNA replication (Fig. 6c), suggesting that S-phase onset does not play a role in the observed downregulation of the M and G1 clusters during a normal cell cycle. We also noted that the capacity of these cells to undergo S phase appeared to be independent of prior activation of the MBF-dependent program, which occurred during and peaked after bulk DNA replication. It therefore likely relied on either the basal expression of MBF targets during the extended G1 block in these experiments or the perdurance of essential factors. Finally, a significant increase in the expression of the S-phase histone gene *hht1* was observed during the initial G1 block (Fig. 6e), as expected from its dependency on low Cdk1 activity (Fig. 4d). This was followed by a reduction in *hht1* transcript levels upon reaching high Cdk1 activity and eventually another increase, likely due to the degradation of the synthetic Cdk1 module by the APC at mitotic exit. Similar results were obtained when simultaneous S and M were induced from G2 cells that were first reset into G1 without an intervening mitosis^16^ (Supplementary Fig. 3a–e). These data suggest that even when cell cycle progression is rewired to the extent that M and S overlap, a normal sequence of periodic gene expression is triggered upon experiencing mitotic Cdk1 activity. Undergoing DNA replication therefore has no direct impact on the Cdk1-driven oscillations in transcription.

To extend these conclusions, we performed RNA sequencing in the G1 arrest/release setup (Fig. 6a), with samples taken at 0, 20 and 40 min (Fig. 6f). Strikingly, as observed for *ace2*, a significant part of the mitotic cluster was strongly induced at 20 min and downregulated by 40 min (Fig. 6f and Supplementary Data 2). The latter is likely due to activation of the APC negative feedback loop by high Cdk1 activity. Moreover, a majority of G1 genes, including MBF and Ace2 targets, were induced at both time points. However, as anticipated from the *cdc22* qPCR data (Fig. 6d), the expression ratios were on average lower at 40 min (Supplementary Fig. 3f). When analysing the behaviour of S-phase genes, we noted that 44% made a standard 1.5 cutoff at 20 min and 69% at 40 min. A significant part of this cluster is therefore also induced in these experiments. Interestingly, changes in *hht1* in the qPCR data and more generally of the histone genes in the RNA-seq data were limited and often below the cutoff. This suggests that distinct subsets of the S cluster may be subject to differential regulation. Finally, a significant fraction of the G2 genes were repressed in the T0 to 20 min comparison (>1.5-fold decrease, 36%), when cells undergo mitosis and DNA replication. Interestingly, this indicates that rapid exposure to high Cdk1 activity can repress the G2 cluster during mitosis, while cells are expressing M genes. The repression of the G2 cluster was somewhat attenuated by 40 min (Fig. 6f), probably due to a reduction in Cdk1 activity at mitotic exit. This reflects the behaviour of *spd1* and suggests that expression of G2 genes requires entry into G2 (Fig. 4e) but is repressed by high Cdk1 activity at mitotic onset.

We conclude that the absence of strong interference between the onset of cell cycle transitions and transcriptional oscillations is an underlying feature of the periodic transcriptional program in fission yeast.

## Discussion

Progression through the eukaryotic cell cycle requires the coordination of a host of pathways that operate in an orderly manner to robustly sustain the sequence of cell cycle events. In particular, temporal regulation of the expression of cell cycle components, including phase-specific Cdk1 substrates, plays a central role in this process. Although the periodic expression of distinct gene clusters at each cell cycle stage has been established, little is known about the networks that link cell cycle progression with transcriptional oscillations. Interestingly, the possibility to re-wire the fission yeast cell cycle, bypassing phases by externally altering Cdc2/Cdk1 activity^16^, suggests that critical Cdk1 substrates are either constantly present, allowing for rapid response to Cdk1 activity changes, or expressed in coordination with the different Cdk1-dependent cell cycle transitions. In this work, we propose a novel model for the orchestration of cell cycle processes in which both the transitions through the major phases and the periodic expression of cell cycle genes are directly dependent on the same profile of quantitative changes in Cdk1 activity.

In contrast to budding yeast, where periodic gene expression still occurs in arrested cells^6^,^7^,^8^, our data demonstrate that the transcriptional network that operates in fission yeast is associated with cell cycle progression. It has been suggested that the Rad53-dependent S-phase checkpoint can alter periodic transcription in budding yeast^21^. However, in the minimal system, it was previously shown that the DNA replication checkpoint is not activated in G1 or G2-arrested cells, excluding a role for this pathway in the observed inhibition of transcriptional oscillations^16^. Importantly, our study reveals that periodic expression is in fact not linked to the completion of cell cycle phases *per se*: quantitative changes in a single qualitative Cdk1 activity are sufficient to control the transcriptional oscillations even when cells are prevented from undergoing cell cycle transitions.

We observe that the mitotic genes, including PBF targets, are activated only when cells are exposed to high Cdk1 activity. Interestingly, this was also true for the expression of MBF targets (for example, *cdc18* and *cdc22*). This suggests that full activation of the periodic transcription program associated with DNA replication occurs prior to G1 and S in normal cells, when they commit to mitosis with elevated levels of Cdk1 activity. Mitosis *per se* is not essential for this, as induction of MBF targets is still observed when this event is bypassed, although to a lesser extent. This attenuated response likely results from an intermediate Cdk1 activity (between the S and M thresholds) in cells released in lower inhibitor concentrations (Fig. 5). In contrast, induction of the S phase histone genes is dependent on low Cdk1 activity. Again, their expression can be induced even in mitotic cells by simply inhibiting Cdk1. This may optimize the coupling of periodic transcription with genome duplication, as Cdk activity must first be strongly reduced for replication origin licensing and then increased to pass the S phase threshold. Finally, we also uncovered that cell cycle transitions do not interfere with the transcriptional program imposed by Cdk1 activity. These findings therefore support a model in which periodic oscillations in gene expression can be generated from any point in the cell cycle, provided that cells are exposed to the proper levels of Cdk1 activity.

Our results also imply that key aspects of periodic transcription in fission yeast are set by the drastic and unique changes in Cdk1 activity that occur between mitotic onset (high activity, induction of PBF and MBF targets) and mitotic exit (low activity resulting from cyclin B degradation, induction of S genes). The delay between PBF and MBF target expression could be a consequence of a difference in sensitivity to Cdk1 activity. PBF may be more sensitive to phosphorylation by Cdk1 and therefore activated earlier than MBF, with a direct impact on the timing of expression of their respective targets. Alternatively, this may rely on a timer activated at high Cdk1, although the potential mechanisms involved remain unknown.

Our observation that mitosis serves as the central node for establishing periodic transcription also raises the question of feedback regulation from the expression of specific clusters. Indeed, mutations in transcription factors controlling M genes^22^,^23^,^24^ or alteration of the RNA polymerase II Mediator complex^25^ have an impact on mitotic entry^26^,^27^. This may provide additional layers of regulation to ensure the proper sequence of cell cycle events, together with the quantitative dependence of the G1/S and G2/M transitions on Cdk1 activity levels. Interestingly, the Mediator complex is specifically recruited to both PBF and MBF target genes in early mitosis^27^, and it may thus play a role in linking periodic transcription with the control of Cdk1 activity.

Our findings provide a new understanding of the regulation of cell cycle progression and periodic transcription. Consistent with previous work in fission yeast^16^,^28^ and the accumulating evidence in other systems for an important quantitative component in the control of cell proliferation^17^,^18^,^19^, the conclusions presented here strongly argue that qualitative differences in Cdk activity (that is, activities mediated by distinct cyclins) are not essential for core cell cycle control. Not only can cell cycle transitions be triggered by changes in the levels of a single Cdk activity, but we now show that the underlying organization of the expression landscape that is essential for cell proliferation is also dependent on such changes. This suggests that the events associated with cell proliferation in eukaryotes may function at two levels. Basic cell cycle progression may rely on simple, quantitative inputs from Cdks, without a significant contribution from cyclin specificity. On the contrary, responding to challenging environments may require Cdk1 to associate with different cyclins. Thus, the complexity observed in wild-type cells may have evolved as a response to changing conditions, when a purely quantitative operation of cell cycle control and periodic transcription may not have been sufficient to sustain efficient cell proliferation. Taken together, our results give rise to a novel model for how eukaryotic cells may orchestrate gene expression with the control of the major cell cycle regulators during growth and division, which may provide a new perspective for future investigation of these processes in higher eukaryotes.

## Methods

### Fission yeast strains and methods

The *S. pombe* strains used in this study were DC240 (*leu1Δ::Pcdc13::cdc13-L-cdc2as::cdc133'UTR::ura4+ cdc2Δ::kanMX6 cdc13Δ::natMX6 cig1Δ::ura4+ cig2Δ::ura4+ puc1Δ::ura4+ ura4-D18 h+*)^16^, DC510 (*leu1Δ::Pcdc13::cdc13-L-cdc2as::cdc133'UTR::ura4+ cdc2Δ::kanMX6 cdc13Δ::natMX6 cig1Δ::hphMX6 cig2Δ::kanMX6 puc1Δ::leu2+ nda3-km311 ura4-D18;* this study) and CG179 (*nda3-km311*)^29^. DC240 was previously described^16^. DC510 has been generated for this study and is a derivative of DC240 in which the deletions of *cig1, cig2* and *puc1* are complete deletions of the open reading frames obtained by homologous recombination. The *nda3-km311* cold-sensitive mutation has been previously described^29^. All experiments were carried out in minimal medium plus supplements (EMM6S)^30^,^31^ at 32 °C except where otherwise noted. The 3-MBPP1 inhibitor (A602960, Toronto Research Chemicals Inc.) was dissolved in dimethylsulphoxide at a stock concentration of 10 mM and added to cultures at the indicated concentrations. Release from inhibitor-induced blocks was achieved by filtration and extensive washing in pre-warmed medium.

### Assessment of cell cycle progression

For determining the percentage of binucleated cells in the population (mitotic index), cells were heat-fixed for 2 min at 70 °C on glass slides and stained with a 1:1 DAPI (1 μg ml^−1^): Blankophor (MP Biochemicals) solution. This stained the DNA as well as the cell wall and division septum, allowing the scoring of both binucleated cells and aberrant nuclei (‘cut' cells). Imaging was performed using a Zeiss Axio Observer (Carl Zeiss Inc.) equipped with a Lumencor Spectra X illumination system and a Hamamatsu Orca Flash 4.0V2 sCMOS camera.

DNA content analyses were performed by flow cytometry. Cells were fixed in 70% ethanol, washed in 50 mM sodium citrate, treated with RNase A (0.1 mg ml^−1^) and stained with propidium iodide (2 mg ml^−1^). DNA content was determined using a BD Accuri C6 flow cytometer. The fission yeast cell cycle has a short G1, with S phase occurring prior to cytokinesis. This has important consequences on the interpretation of flow cytometry profiles. As a result of the short G1, cells have a 2C DNA content for most of their cell cycle. In synchronized populations, a transient 4C peak is observed when S phase occurs (cells are then binucleated), which is then resolved when cells undergo cytokinesis. This 4C peak is almost undetectable in asynchronous wild-type cells as it only represents a small percentage of the entire population. A larger 4C fraction can result from cytokinesis defects or re-replication without an intervening mitosis. Conversely, appearance of a 1C peak reflects an elongation of G1 or a delay in S phase, leading to cytokinesis occurring prior to DNA replication. Finally, cell size can have a significant effect on the cytometry profiles, as non-nuclear staining by propidium iodide increases with size. The profiles become slightly shifted to the right in larger cells and to the left in smaller cells, despite identical nuclear DNA contents.

### RNA methods and quantitative PCR

Total RNA was isolated using the RNeasy kit (Qiagen), following the manufacturer's protocol, or using the hot phenol method^32^. Briefly, cell pellets were resuspended in 500 μl TES solution (10 mM Tris–HCl, pH 7.5, 10 mM EDTA, 0.5% SDS), transferred to screw-capped tubes and 500 μl acid phenol was added. Samples were then vortexed vigorously prior to incubation at 65 °C for 60 min with occasional, brief vortexing. After incubation on ice for 5 min, samples were centrifuged at high speed for 5 min. The upper aqueous phase was saved and extracted two times with 500 μl chloroform. Total RNA was then precipitated by sodium acetate (3M, 1/10 volume) and ethanol (2.5 volume) and washed with 70% ethanol. The RNA pellets were then resuspended in 100 μl RNase-free water and purified using the RNeasy kit (Qiagen), according to the manufacturer's instructions. RNA isolation was followed by DNase treatment to eliminate DNA contamination, using the Turbo DNA-free kit (Ambion), according to the manufacturer's recommendations. RNA concentrations were determined and ∼1 μg of total RNA was reverse-transcribed using the iScript cDNA synthesis kit (Bio-Rad). A volume of 2 μl of a 1:10 dilution of the complementary DNA reactions were used for qPCR analyses on a Bio-Rad CFX96 instrument. Relative fold changes were normalized to actin RNA levels. All measurements were performed in duplicate using samples from at least two biological repeats. Primers for the qPCRs can be found in Supplementary Table 3.

### Microarray and RNA sequencing

RNA samples (5–30 μg) from the indicated time points were used for microarray analysis (BEA, Karolinska Institute) or for RNA sequencing (BGI, Hong Kong). For microarray experiments, expression values from two biological repeats were averaged and analysed with the ArrayStar (DNASTAR) software. For RNA sequencing, quality assessment of the sequence reads was performed by generating QC statistics with FastQC (http://www.bioinformatics.bbsrc.ac.uk/projects/fastqc). RNA-seq reads were then mapped to the *S. pombe* 972 *h*- genome from NCBI Tophat^33^. Cufflinks^34^ was used for transcript assembly of individual samples, and the corresponding gene counts were obtained with HTSeq (http://www-huber.embl.de/users/anders/HTSeq/doc/overview.html). Samples from four biological repeats were pooled to create two independent RNA sequencing samples, and DESeq^35^ was then used to identify differentially expressed genes. Heatmaps were generated in R using the hierarchical clustering method.

## Additional information

**Accession codes:** The microarray and RNA sequencing data can be retrieved under the GSE70717 accession number.

**How to cite this article:** Banyai, G. *et al.* Cdk1 activity acts as a quantitative platform for coordinating cell cycle progression with periodic transcription. *Nat. Commun.* 7:11161 doi: 10.1038/ncomms11161 (2016).

## Supplementary Material

Supplementary Information Supplementary Figures 1-3, Supplementary Tables 1-3 and Supplementary Reference

Supplementary Data 1 Periodic gene expression in G2-arrested cells

Supplementary Data 2 Periodic gene expression in cells undergoing simultaneous S and M
