# Supplementary material for: Cdk1 activity acts as a quantitative platform for coordinating cell cycle progression with periodic transcription
Source: Nat Commun. 2016 Apr 5;7:11161. doi: 10.1038/ncomms11161 (PMC4822045; doi:10.1038/ncomms11161)
Supplement: Supplementary Information — Supplementary Figures 1-3, Supplementary Tables 1-3 and Supplementary Reference [file ncomms11161-s1.pdf]

Supplementary Figure 1

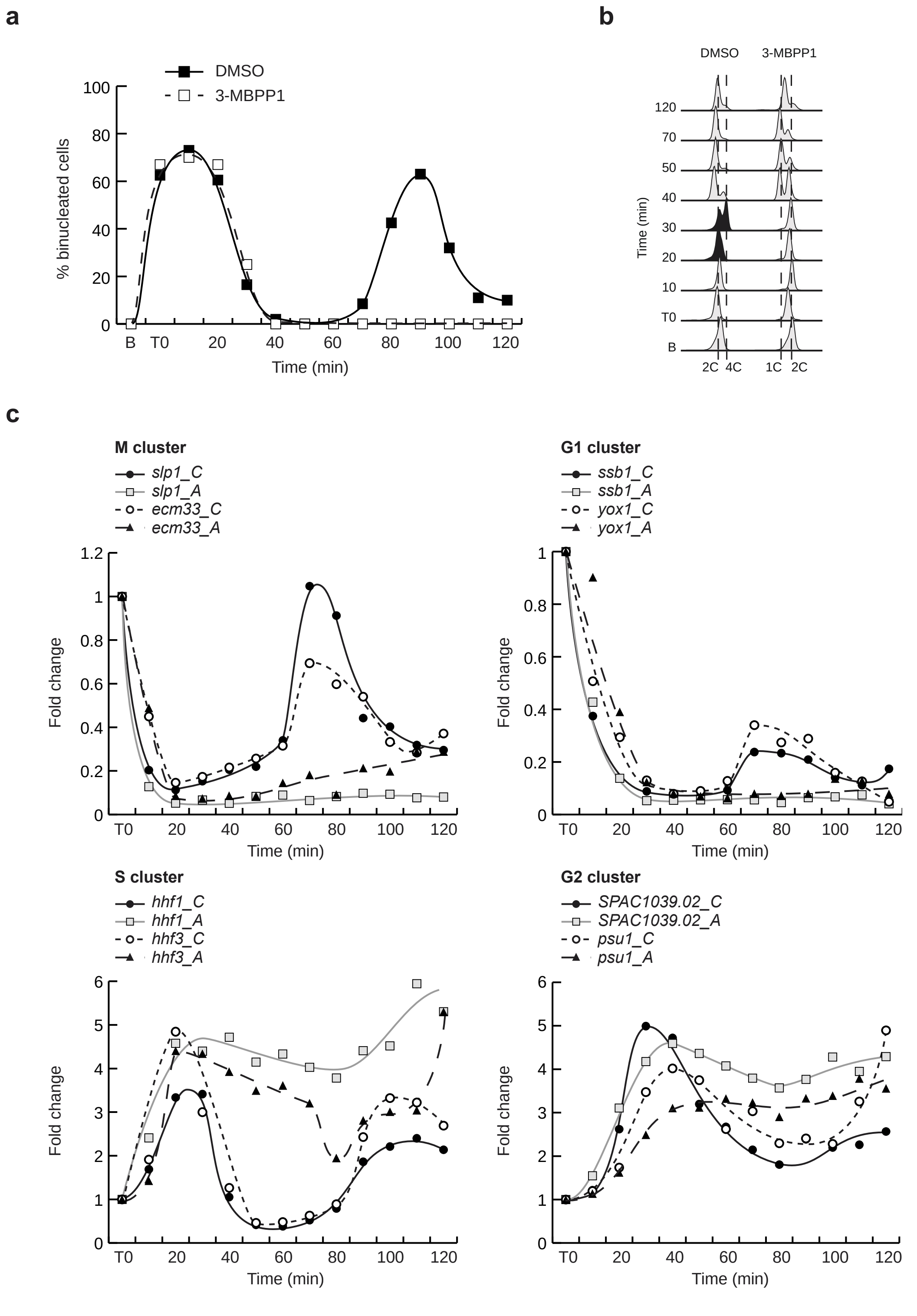

Supplementary Figure 1. Periodic transcription in G1-arrested fission yeast cells
Cells were treated as in Figure 1a. **a.** Percentage of binucleated cells. Both control and inhibitor-treated cells initially undergo mitosis. However, cells subsequently treated with inhibitor at T0 remain blocked in G1 (see b) and do not progress to the next mitosis. n>100 at each time point. Note that differences in the duration of the wash during these experiments can induce negligible variations in the timing of the first peak of binucleated cells (compare with Fig. 1b). **b.** DNA content analysis of cells in *a*. While control cells progress through S phase (black profile) and cytokinesis, inhibitor-treated cells accumulate in G1 prior to S phase with a 1C DNA content (see Methods section for the interpretation of flow cytometry profiles in fission yeast). **c.** Changes in gene expression for the indicated genes in *a*. *C* and *A* refer to cycling (DMSO-treated) and arrested (inhibitor-treated) cells, respectively. Fold changes are normalised to actin RNA levels and represented relative to the values at T0 (set to 1). Consistent with our model, the periodicity of the G2 genes is lost in inhibitor-treated cells, although we observe an initial increase in transcription that is not detected with *spd1* (Fig. 1; this may result from different levels of sensitivity to Cdk1 activity).

Supplementary Figure 2

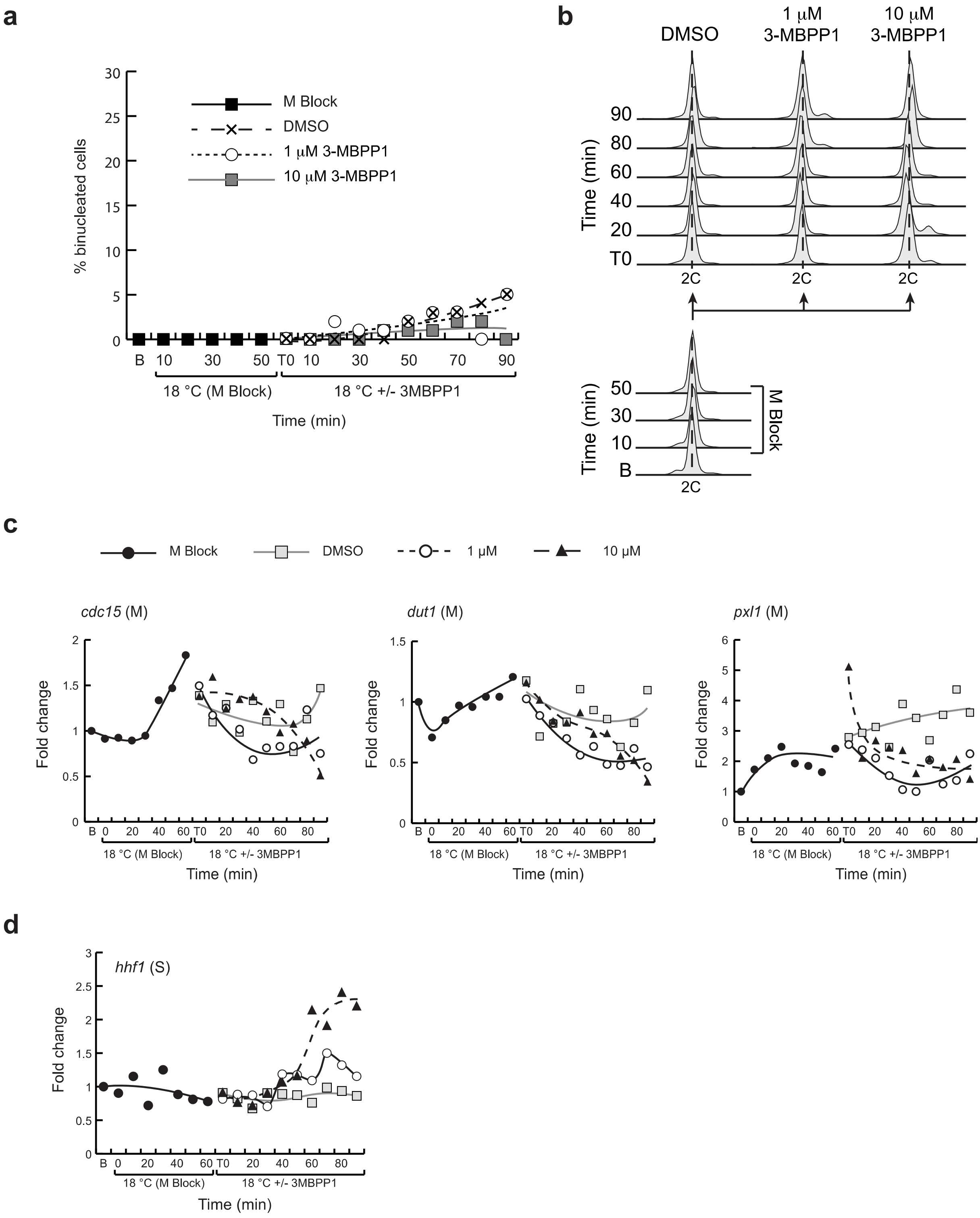

Supplementary Figure 2. Periodic transcription and Cdk1 activity levels
a. Percentage of binucleated cells for the experiments in Fig. 4. n>100 at each time point. **b.** DNA content analysis for the experiments in Fig. 4. In control and inhibitor-treated cultures, cells remain blocked in mitosis without changes in DNA content. **c, d.** Changes in expression of the indicated genes as in Fig. 4. Fold changes are normalised to actin RNA levels and represented relative to the values at B (set to 1, see Fig. 4a). Legends in *d* are as in *c*.

Supplementary Figure 3

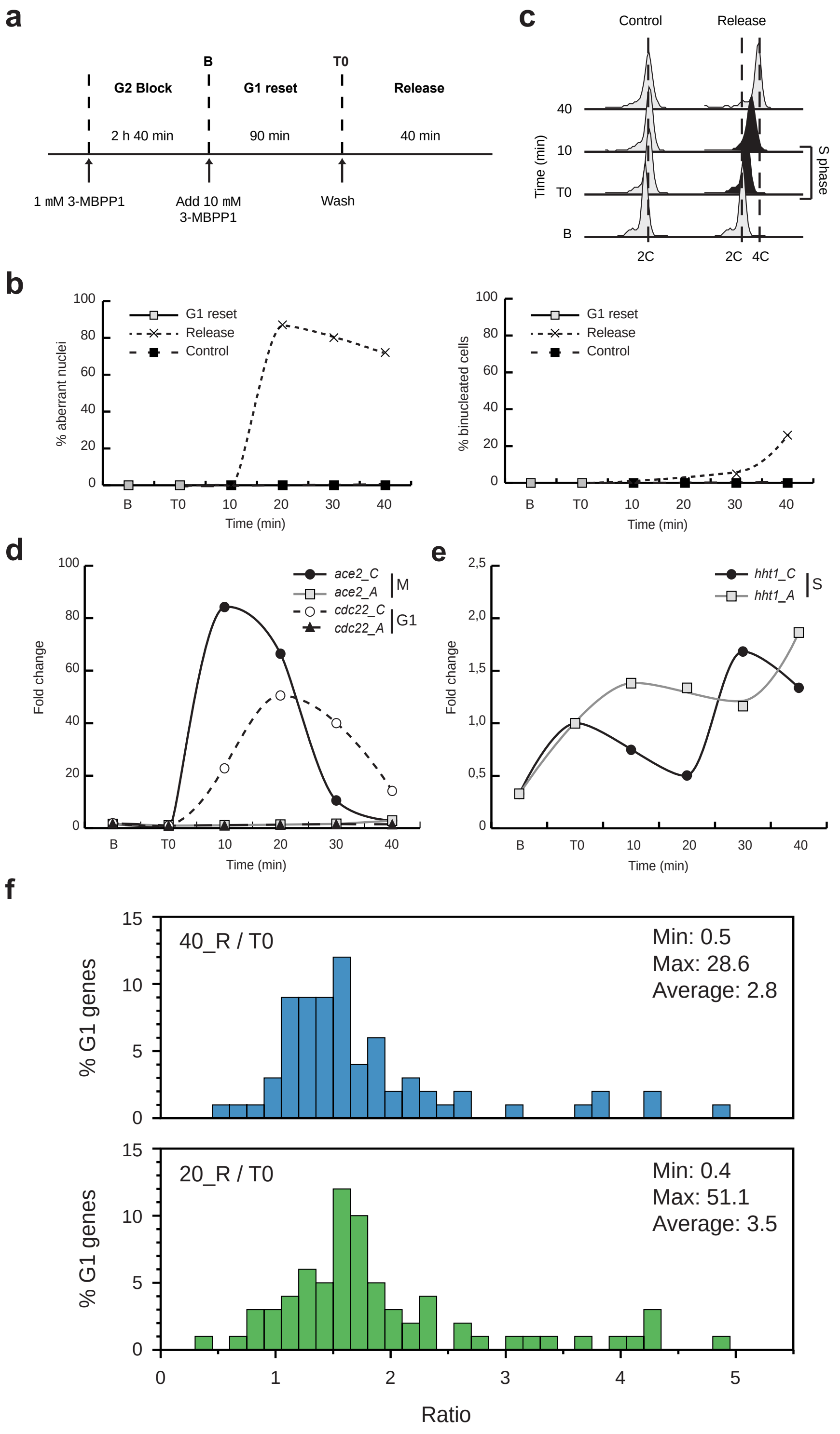

Supplementary Figure 3. Interplay between cell cycle transitions and periodic transcription
a. Schematic representation of the experimental procedure. Inhibitor-sensitive minimal cells were synchronised in G2 by addition of 1 µM 3-MBPP1 for 2 h 40 min. The cultures were then treated with 10 µM 3-MBPP1 for 90 min to induce a G1-reset without an intervening mitosis[1]. Subsequent release from the high inhibitor block at T0 (culture washed with DMSO-containing medium, as compared with control cells maintained in 10 µM 3-MBPP1) induces overlapping S and M phases[1]. Samples were collected at the indicated time points for assessing changes in gene transcription. **b.** Percentages of aberrant nuclei (left panel) and binucleated cells (right panel) as in Fig. 6. n>100 at each time point. **c.** DNA content analysis of cells in *a*. Released cells undergo rapid re-replication (black profiles) while the control, blocked cells remain in a G1-like phase with a 2C DNA content. **d, e.** Changes in gene expression for the indicated genes during the release in *a*. *R* and *A* refer to released (DMSO-washed) and arrested (maintained in 10 µM inhibitor after T0) cells, respectively. Fold changes are normalised to actin RNA levels and represented relative to the values at T0 (set to 1). **f.** Distribution of the expression ratios (20_R/T0 and 40_R/T0) for the G1 genes from Fig. 6f. For viewing purposes, genes with ratios over 5 are excluded from the histograms (9 out of 81 genes for each time point). Minimal, maximal and average values are indicated. Histogram steps are 0.15.

Supplementary Tables

Supplementary Table 1. Differences in periodic gene expression between cycling and G2-arrested cells

		1.5 cut-off			1.15 cut-off		
		**20_C/20_A**	**60_C/60_A**	**20_A/60_A**	**20_C/20_A**	**60_C/60_A**	**20_A/60_A**
**M**	**High**	53	53	44	82	78	91
	**Medium**	9	20	24	22	44	71
	**Low**	4	18	24	26	47	64
**G1**	**High**	0	74	37	15	89	85
	**Medium**	0	48	22	4	81	59
	**Low**	0	26	15	8	74	41
**S**	**High**	0	92	8	0	100	54
	**Medium**	0	62	8	8	92	31
	**Low**	0	23	8	0	62	38
**G2**	**High**	0	10	2	2	33	19
	**Medium**	0	8	4	2	19	40
	**Low**	0	3	2	2	22	17

Percentages of genes above the indicated cut-offs. High, medium and low refer to the groups of induction amplitudes in Fig. 2g.

Supplementary Table 2. Changes in periodic gene expression in cells undergoing simultaneous S and M

	1.5 cut-off	
	**20 / T0**	**40 / T0**
**M**	64	23
**G1**	71	60
**S**	44	69
**G2**	12	12

Percentages of genes above the 1.5 cut-off in Fig. 6f.

Supplementary Table 3. Primers used in this study

Primer name	Sequence
act1RTF	CCCAAATCCAACCGTGAGAAG
act1RTR	TGTGGGTAACACCATCACCAGAG
ace2RTF	CCTCCTTTGATGCTTCCTTATCAC
ace2RTR	TGTTGAGAGTTGGTCAAGGAAAATG
slp1RTF	GAGTTGGTTGTCTCTCCTGGAATC
slp1RTR	CACTAGAGTGTCCCTGAAGAGTCCC
cdc15RTF	TCTCATAATGCCGAGACTGAG
cdc15RTR	GTAGCAGTAGGTTGAAGTTGAC
ecm33RTF	GTAAACTTTCTAACGTTACCACTG
ecm33RTR	GTGTTAGAGAAATACAAGTTACCAG
dut1RTF	CTGAATGTATAGTTCCAAGAAGAG
dut1RTR	GCGCCAGTGTCAATGCTATG
cdc18RTF	CCGTCCTATAATTCTACTGCCAAATTG
cdc18RTR	TTGACGAAAGAAAGATTCCACAATTG
cdc22RTF	CTAAACACGGAATTCGTAATTCTTTGTTAG
cdc22RTR	GTTAGAAGTATATGGCTCAAAACACTCATTG
ams2RTF	CCTGTCAACATAAACGGTCTTTC
ams2RTR	CATGGAACAAAGAATCTAACTCAAG
eng1RTF	GTCTATCTGGTGATTCCTTGGC
eng1RTR	TTAACCCATTCCTTATTGTTCCCAG
hht1RTF	GGAGGTGTTAAGAAGCCTCATCG
hht1RTR	ACCAAACGTTGGAAAGGTAGCTTAC
hta1RTF	GCCGCCGTTTTGGAATATTTG
hta1RTR	GCGAGTTGAAGATGACGGG
hhf1RTF	GCTAAGCGTCACCGTAAAATTC
hhf1RTR	GCTTGAGAACAGCACGAGTC
spd1RTF	CATTCAAGGCTCCTTAATGGACG
spd1RTR	GTGTTGTAAAGCGGTGGGTTATAGG
hhf3RTF	CAAGGTATTACTAAGCCTGCC
hhf3RTR	GCTTGGCATGCTCAGTATAG
SPAC1039.02RTF	CTCTTCGCAACGTTAACGATTATC
SPAC1039.02RTR	GGTATGGACGGTGTCATCGC
psu1RTF	CTTCTGCTCAATACTATGTTAACAAG
psu1RTR	CATACCGGCACCAAAGACAAG
ssb1RTF	CCTCAATACCGTTATATTATAACTATTG
ssb1RTR	CGACGTCGTCAAAGACGTTG
yox1RTF	GCGAAACTTCTACAGGACAGG
yox1RTR	ATGAATCATTTCGCCTACGACAG

Supplementary Reference

1. Coudreuse, D. & Nurse, P. Driving the cell cycle with a minimal CDK control network. *Nature* **468,** 1074–1079 (2010).
